# Supplementary material for: Apportionment and Spatial Pattern Analysis of Soil Heavy Metal Pollution Sources Related to Industries of Concern in a County in Southwestern China
Source: Int J Environ Res Public Health. 2022 Jun 16;19(12):7421. doi: 10.3390/ijerph19127421 (PMC9223715; doi:10.3390/ijerph19127421)
Supplement: Supplementary file 1 [file ijerph-19-07421-s001.zip › ijerph-1667334-supplementary.pdf]

Supplementary material

for

Apportionment and Spatial Pattern Analysis of Soil Heavy Metal Pollution Sources Related to Industries of Concern in a County in Southwestern China

Xiaohui Chen [1,2], Mei Lei [1,2,*], Shiwen Zhang [3], Degang Zhang [1,2], Guanghui Guo [1] and Xiaofeng Zhao [1,2]

1 Center for Environmental Remediation, Institute of Geographic Sciences and Natural Resources Research, Chinese Academy of Sciences, Beijing 100101, China; luokeangcxh@163.com (X.C.); zdg_biology2@126.com (D.Z.); guogh@igsnrr.ac.cn (G.G.); zhaoxf2011@163.com (X.Z.)
2 University of Chinese Academy of Sciences, Beijing 100049, China
3 School of Earth and Environment, Anhui University of Science and Technology, Huainan 232001, China; shwzhang@aust.edu.cn
* Correspondence: leim@igsnrr.ac.cn

Table S1 The results of field survey for the soil pollution sources in study area

Industry Classification	Pollution Sources	Area(ha)	the main migration pathways for soil contamination
Nonferrous metal mining	Mining sites	535.96	dry and wet deposition of heavy metals from mining and transportation
	Concentrators	846.03	dry and wet deposition of heavy metals from concentrating and transportation
	Tailings ponds	1618.41	surface runoff and dust from tailings, espatially left over from history
Nonferrous metal smelting	Smelters	837.26	dry and wet depostion of heavy metals from smelting
	Slag heaps	98.67	surface runoff and dust from slag

Table S2 Descriptive statistics of soil heavy metals based on different land use

Land use		As (mg kg^{-1})	Cd (mg kg^{-1})	Pb (mg kg^{-1})	Zn (mg kg^{-1})	Ni (mg kg^{-1})	Cr (mg kg^{-1})	Sb (mg kg^{-1})	Cu (mg kg^{-1})
Dry land (n=412)	Mean	222.53	2.84	328.4	514.12	98.46	142.32	7.25	256.17
	SD	102.13	1.52	177.52	140.12	43.52	62.32	5.92	128.13
	Samples surpassed RSV(%)	92.32	100.00	80.12	62.54	18.34	10.13	0.00	82.67
Paddy land (n=123)	Mean	215.67	2.56	212.58	411.29	85.62	139.12	8.09	266.58
	SD	99.13	0.98	156.13	150.16	14.15	42.58	3.12	111.38
	Samples surpassed RSV(%)	97.14	100.00	72.14	45.23	18.12	0.00	0.00	87.55
Garden plot (85)	Mean	189.11	2.18	305.12	498.15	78.15	138.14	7.03	198.12
	SD	72.13	0.88	162.34	133.24	10.12	42.12	2.16	93.24
	Samples surpassed RSV(%)	82.22	100.00	74.58	49.34	9.13	5.18	0.00	70.52
RSV(Paddy land)		30	0.4	100	200	70	250	36	50
RSV(other land)		40	0.3	90	200	70	150	36	50

Note: SD, Standard deviation; RSV, Risk screening values based on MEP-PRC and WTO[1,2].

Table S3 Pearson coefficients for heavy metals, major elements and the environmental factors of the two subregion

	coefficient	As	Cd	Pb	Zn	Ni	Cr	Sb	Cu	K	Ca	Mn	Fe	Dist	HI	EL
Subregion 1 (R1)	As	1														
	Cd	0.85**	1													
	Pb	0.53**	0.51**	1												
	Zn	0.53**	0.50**	0.71**	1											
	Ni	0.31**	0.26**	0.32**	0.25**	1										
	Cr	0.39**	0.42**	0.26**	0.26**	0.38**	1									
	Sb	0.57**	0.61**	0.34**	0.28**	0.35**	0.31**	1								
	Cu	0.65**	0.57**	0.50**	0.50**	0.25**	0.25**	0.36**	1							
	K	0.36**	0.27**	0.35**	0.21**	0.47**	0.22**	0.43**	0.36**	1						
	Ca	0.04	0.01	-0.03	-0.02	0.05	-0.02	-0.01	-0.04	0.02	1					
	Mn	0.26**	0.29**	0.35**	0.35**	0.31**	0.23**	0.32**	0.28**	0.41**	-0.01	1				
	Fe	0.26**	0.18**	0.21**	0.12**	0.36**	0.17**	0.32**	0.26**	0.76**	0.002	0.32**	1			
	Dist	-0.27**	-0.23**	-0.31**	-0.31**	-0.11*	-0.07	-0.08	-0.32**	-0.20**	0.14**	-0.13**	-0.13**	1		
	HI	-0.09*	-0.14**	-0.08	-0.06	-0.07	-0.05	-0.09*	-0.05	-0.08	-0.08	-0.10*	-0.03	0.01	1	
	EL	-0.003	0.06	-0.04	-0.01	0.01	0.02	0.07	-0.05	-0.12**	0.04	0.11*	-0.09	0.21**	-0.11*	1
Subregion 2 (R2)	As	1														
	Cd	0.67**	1													
	Pb	0.36**	0.50**	1												
	Zn	0.31**	0.47**	0.80**	1											
	Ni	0.19*	0.25**	0.14	0.11	1										
	Cr	0.36**	0.43**	0.54**	0.47**	0.38**	1									
	Sb	0.36**	0.42**	0.48**	0.39**	0.06	0.39**	1								

coefficient	As	Cd	Pb	Zn	Ni	Cr	Sb	Cu	K	Ca	Mn	Fe	Dist	HI	EL
Cu	00.35**	00.31**	00.46**	00.32**	00.11	00.31**	00.55**	1							
K	00.36**	00.16*	00.14	-00.02	00.10	00.17*	00.31**	00.37**	1						
Ca	00.28**	00.15	00.24**	00.12	-00.02	00.17*	00.23**	00.41**	00.70**	1					
Mn	00.25**	00.15	00.13	00.02	00.07	00.13	00.23**	00.31**	00.68**	00.59**	1				
Fe	00.31**	00.23**	00.18*	00.10	00.04	00.24**	00.31**	00.30**	00.58**	00.55**	00.60**	1			
Dist	-00.19*	-00.41**	-00.35**	-00.46**	-00.13	-00.22**	-00.14	00.01	00.17*	00.09	00.09	-00.05	1		
HI	-00.06	-00.03	-00.08	-00.07	-00.05	00.01	-00.01	-00.14	-00.09	-00.05	-00.12	-00.06	-00.08	1	
EL	00.17*	00.08	00.12	-00.04	00.24**	00.22**	00.15	00.32**	00.29**	00.39**	00.19*	00.19*	00.14	-00.19*	1

Note: ** indicates a significant level of correlation at the 00.01 level (double-tailed), while * is 00.050.

Table S4 Comparison of means between different directions for soil heavy metals mg kg^{-1}

Subregion	Directions	East	South	West
R1	South	As(51.17*) ; Cd(0.46*)<br>Pb(34.47*) ; Zn(-3.06)<br>Ni(6.23) ; Cr(22.43*)<br>Sb(1.23) ; Cu(31.54)		
	West	As(-7.54) ; Cd(0.08)<br>Pb(-54.90*) ; Zn(-73.06*)<br>Ni(-7.11) ; Cr(13.59)<br>Sb(0.02) ; Cu(-6.06)	As(-58.71*) ; Cd(-0.38*)<br>Pb(-89.38*) ; Zn(-69.99*)<br>Ni(-13.34*) ; Cr(-8.84)<br>Sb(-1.21) ; Cu(-37.61*)	
	North	As(30.46*) ; Cd(0.57*)<br>Pb(-32.75) ; Zn(-49.97*)<br>Ni(-6.56) ; Cr(21.45)<br>Sb(1.62) ; Cu(27.73)	As(-20.70) ; Cd(0.11)<br>Pb(-67.22*) ; Zn(-46.91*)<br>Ni(-12.80) ; Cr(-0.98)<br>Sb(0.39) ; Cu(-3.81)	As(38.00*) ; Cd(0.49*)<br>Pb(22.15) ; Zn(23.09)<br>Ni(0.54) ; Cr(7.86)<br>Sb(1.60) ; Cu(33.79*)
R2	South	As(133.59*) ; Cd(0.71)<br>Pb(97.63*) ; Zn(15.88)<br>Ni(20.53*) ; Cr(41.43*)<br>Sb(2.81*) ; Cu(118.48*)		
	West	As(39.33) ; Cd(-0.33)<br>Pb(-2.39) ; Zn(-34.98)<br>Ni(22.24*) ; Cr(9.37)<br>Sb(-0.42) ; Cu(-17.16)	As(-94.26*) ; Cd(-1.04*)<br>Pb(-100.00) ; Zn(-50.86)<br>Ni(1.71) ; Cr(-32.06)<br>Sb(-3.23*) ; Cu(-135.65*)	
	North	As(37.55) ; Cd(-0.07)<br>Pb(63.48) ; Zn(-6.33)<br>Ni(31.50*) ; Cr(24.60)<br>Sb(1.05) ; Cu(43.53)	As(-96.03*) ; Cd(-0.78)<br>Pb(-34.15) ; Zn(-22.21)<br>Ni(10.97) ; Cr(-16.84)<br>Sb(-1.76) ; Cu(-74.95)	As(-1.77) ; Cd(0.26)<br>Pb(65.87) ; Zn(28.65)<br>Ni(9.26) ; Cr(15.23)<br>Sb(1.47) ; Cu(60.69)

Note: * indicates a significant level of differences between means at the 0.05 level;
East means the nearest pollution source is on the east of sample, and so forth.

Table S5 Rule of thumb for interpreting the size of a correlation coefficient [3]

Size of Correlation	Interpretation
0.90 to 1.00 (−0.90 to −1.00)	Very high positive (negative) correlation
0.70 to 0.90 (−.70 to −0.90)	High positive (negative) correlation
0.50 to 0.70 (−0.50 to −0.70)	Moderate positive (negative) correlation
0.30 to 0.50 (−0.30 to −0.50)	Low positive (negative) correlation
0.00 to 0.30 (0.00 to −0.30)	negligible correlation

Table S6 Background values of soil heavy metals in study area mg/kg

As	Cd	Cu	Pb	Zn	Sb
18.4	0.218	46.3	40.6	89.7	2.44

Figure S1 Auto-variograms and cross-variograms between the heavy metals extracted by PMF

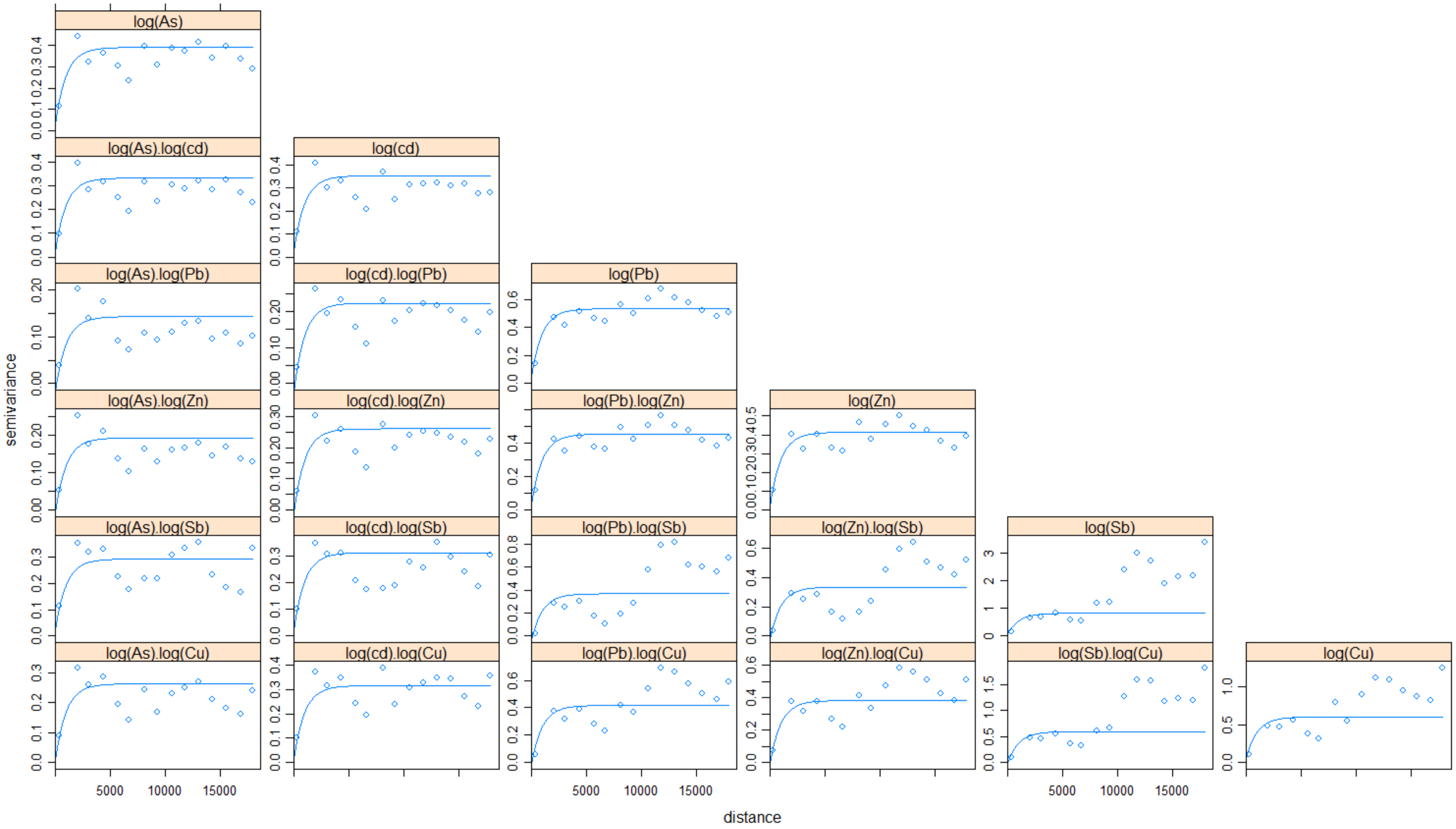

References

1. Chang, A.C.; Page, A.L.; Asano, T.; Hespanhol, I. Developing human health-related chemical guidelines for reclaimed wastewater irrigation. *Water Science and Technology* **1996**, *33*, 463-472, doi:https://doi.org/10.1016/0273-1223(96)00449-0.
2. MEP-PRC. Environmental Quality Standards for Soil (GB15618-2018). **2018**.
3. Mukaka, M.M. Statistics corner: A guide to appropriate use of correlation coefficient in medical research. *Malawi Med J* **2012**, *24*, 69-71, doi:10.2166/wh.2012.000.
